# Mendelian randomization analyses implicate biogenesis of translation machinery in human aging

**DOI:** 10.1101/gr.275636.121

**Published:** 2022-02

**Authors:** Sara Javidnia, Stephen Cranwell, Stefanie H. Mueller, Colin Selman, Jennifer M.A. Tullet, Karoline Kuchenbaecker, Nazif Alic

**Affiliations:** 1 Institute of Healthy Ageing, Research Department of Genetics Evolution and Environment, University College London, London WC1E 6BT, United Kingdom;; 2 Institute of Health Informatics, University College London, London NW1 2DA, United Kingdom;; 3 Institute of Biodiversity, Animal Health and Comparative Medicine, College of Medical, Veterinary and Life Sciences, University of Glasgow, Glasgow G12 8QQ, United Kingdom;; 4 School of Biosciences, University of Kent, Canterbury CT2 7NZ, United Kingdom;; 5 UCL Genetics Institute, Research Department of Genetics Evolution and Environment, University College London, London WC1E 6BT, United Kingdom;; 6 Division of Psychiatry, University College London, London W1T 7NF, United Kingdom

## Abstract

Reduced provision of protein translation machinery promotes healthy aging in a number of animal models. In humans, however, inborn impairments in translation machinery are a known cause of several developmental disorders, collectively termed ribosomopathies. Here, we use casual inference approaches in genetic epidemiology to investigate whether adult, tissue-specific biogenesis of translation machinery drives human aging. We assess naturally occurring variation in the expression of genes encoding subunits specific to the two RNA polymerases (Pols) that transcribe ribosomal and transfer RNAs, namely Pol I and III, and the variation in expression of ribosomal protein (RP) genes, using Mendelian randomization. We find each causally associated with human longevity (β = −0.15 ± 0.047, *P* = 9.6 × 10^−4^, *q* = 0.015; β = −0.13 ± 0.040, *P* = 1.4 × 10^−3^, *q* = 0.023; β = −0.048 ± 0.016, *P* = 3.5 × 10^−3^, *q* = 0.056, respectively), and this does not appear to be mediated by altered susceptibility to a single disease. We find that reduced expression of Pol III, RPs, or Pol I promotes longevity from different organs, namely visceral adipose, liver, and skeletal muscle, echoing the tissue specificity of ribosomopathies. Our study shows the utility of leveraging genetic variation in expression to elucidate how essential cellular processes impact human aging. The findings extend the evolutionary conservation of protein synthesis as a critical process that drives animal aging to include humans.

The changing demographic of human societies, where the proportion of older people is rapidly increasing (Christensen et al. 2009), makes it imperative to understand the basic biology of aging as an avenue for improvement of the health in older people (Partridge et al. 2018). Work in model organisms has identified a number of genes and processes that drive animal aging (López-Otín et al. 2013; Partridge et al. 2020). These often show striking evolutionary conservation, strongly suggestive of their relevance to aging in humans. However, direct evidence for their involvement in human aging is not often sought owing to the inherent methodological limitations, long timescales, and excessive costs associated with studying aging in human populations. The general challenges of translating findings from model organisms to humans are only starting to be overcome, for example, with human population genetic studies or drug trials (Mannick et al. 2018; Deelen et al. 2019; Torres et al. 2021). Validation of candidate processes as drivers of human aging may prove essential to show their potential as targets for treatment of age-related diseases and dysfunctions.

Multiple lines of evidence obtained in model organisms indicate that the biogenesis of protein synthesis machinery is one such conserved processes that drives organismal aging. For example, loss of function in the genes encoding ribosomal proteins (RPs) can extend life span in yeast and worms (Kaeberlein et al. 2005; Chen et al. 2007; Chiocchetti et al. 2007; Curran and Ruvkun 2007; Hansen et al. 2007). Our own work has shown that reducing the activity of Pol I, which supplies most ribosomal RNAs (rRNAs), and Pol III, which generates the 5S rRNA and transfer RNAs (tRNAs), can extend life span in fruit flies, with the effect of Pol III also conserved in worms and yeast (Filer et al. 2017; Martínez et al. 2020). However, the relevance of the biogenesis of translation machinery to human aging has not been examined.

Protein synthesis is essential for cellular and organismal function. Indeed, loss of function in genes required to make ribosomes, the macromolecular machines that synthesize cellular proteins, is a cause of disease in humans (Mills and Green 2017). A range of human developmental disorders results from mutations in genes required for ribosome biogenesis, including RP genes (Mills and Green 2017) and subunits of Pol I and III (Bernard et al. 2011; Dauwerse et al. 2011; Weaver et al. 2015; Paolacci et al. 2018). These disorders are collectively called ribosomopathies, and their occurrence appears to contradict evidence from aging studies in model organisms, where loss of function in genes required for protein synthesis promotes longevity. However, it is possible that human disease–causing mutations cause a substantial loss of function present during development, whereas the prolongevity interventions in model organisms are induced by a more moderate, possibly tissue-specific, reduction in activity in the adult. This idea prompted us to seek ways to assess the impact of moderate reduction in expression of components of translation machinery in adult tissues on human aging.

In this study, we establish a new paradigm for *in homine* validation of candidate aging processes from model organisms. Using Mendelian randomization (MR), we examine if naturally occurring variation in the expression of the subunits of Pol III or Pol I complexes or of RPs in adult humans is causally associated with human longevity. We provide evidence that biogenesis of protein synthesis machinery in specific adult tissues drives human aging.

## Results

### Expression of Pol III–specific subunits in the adipose tissue is linked to human longevity

**Figure 1.**
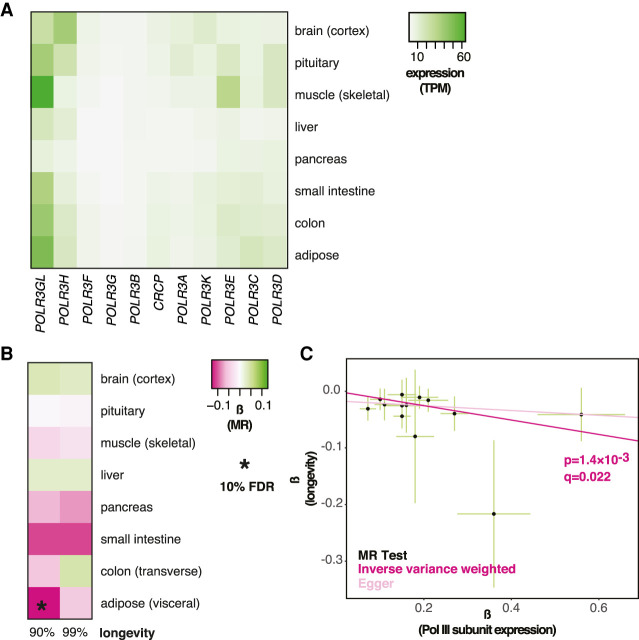
MR analysis of the association between the expression of Pol III–specific subunits and longevity. (*A*) Levels of mRNA coding for Pol III–specific subunits in the indicated tissues are shown. (TPM) Transcripts per million. (*B*) MR analysis was performed using Pol III–specific subunit expression in indicated tissues as exposures and longevity (survival beyond the 90th or 99th percentile) as outcomes. The values of β are given per tissue and the significant associations indicated as those that pass the 10% FDR threshold (accounting for eight tissues and two outcomes, i.e., 16 tests). (*C*) MR scatter plot of association between Pol III expression in visceral adipose and longevity (90th percentile), showing results of two MR methods. Each black point denotes a *cis*-eQTL; green bars show SEM. Longevity β is the natural log of the odds ratio, where lower values indicate a lower chance of surviving beyond the 90th percentile. Expression β is reported as normalized effect sizes (NESs), with larger values indicating higher expression, and refers to expression of multiple genes.

We initially focused on Pol III because limiting expression of its subunits has been shown to affect life span in multiple animal models (Filer et al. 2017). These effects are tissue specific; for example, reducing Pol III subunit expression specifically in the intestine of the fly and the worm is sufficient to extend their life span (Filer et al. 2017). For this reason, we focused on organs and tissues known to modulate life span in model organisms: the brain, pituitary gland, muscle, liver, pancreas, small and large intestine (colon), and adipose tissue (Libina et al. 2003; Bartke and Brown-Borg 2004; Giannakou et al. 2004; Hwangbo et al. 2004; Broughton et al. 2005; Taguchi et al. 2007; Demontis and Perrimon 2010; Templeman et al. 2017; Woodling et al. 2020). Where gene expression has been shown to be highly correlated between similar tissues (The GTEx Consortium 2020), we used only one representative organ (e.g., visceral adipose for adipose). Because of technical constraints of transcriptomic analyses, no information is available on the variation in expression of genes transcribed by Pol III (or Pol I for that matter), such as rRNAs or tRNAs, requiring us to focus on the expression of the genes that code for the polymerase. Pol III is a protein complex composed of 17 subunits, 11 of which are specific to the enzyme (Vannini and Cramer 2012). The expression of these 11 genes was ubiquitous across these tissues (Fig. 1A) as expected from Pol III's fundamental cellular role. Still, the expression levels differed between tissues, with, for example, the pancreas showing the overall lowest expression (Fig. 1A).

To assess how variation in the expression of Pol III subunits may impact human aging, we gathered expression quantitative trait loci (eQTLs) for all the Pol III–specific subunits in the noted tissues from the Genotype-Tissue Expression (GTEx) project (The GTEx Consortium 2020). Because all the subunits participate in a common function, we pooled all the available *cis*-eQTLs associated with the expression of any of the subunits of the Pol III complex for further analysis. Note that this includes a set of paralogs, *POLR3G* and *POLR3GL*, for which recent work has indicated that previously described differences in their cellular roles most likely arise from expression-level differences (Wang et al. 2020). We used these *cis*-eQTLs as instruments in a two-sample MR analysis to assess their causal effects on human longevity. The outcome was based on a recent meta-analysis of genome-wide association studies of human longevity. It included 11,262 individuals (cases) that had survived beyond the 90th percentile in a given human cohort and 25,483 controls (Deelen et al. 2019). The study also assessed associations in 3484 cases that had survived beyond the 99th percentile.

MR revealed Pol III subunit expression in the visceral adipose as causally associated with the likelihood of surviving above the 90th percentile (β = −0.13 ± 0.040, *P* = 1.4 × 10^−3^, *q* = 0.023) (Fig. 1B,C; for per-*cis*-eQTL effect sizes and gene annotation, see Supplemental Fig. S1A; for details of MR results for all tissues, see Supplemental Table S1). This was statistically significant at a 10% false-discovery rate (FDR), accounting for the number of tissues tested and the two case definitions considered as outcomes (i.e., 16 tests). Note that the interpretation of the absolute value of the causal estimate β is complicated by the fact that *cis*-eQTLs for multiple genes were combined in the MR; however, the direction of the association is not, and the negative β revealed that increased Pol III subunit expression within adipose tissue is detrimental for longevity (Fig. 1C). No evidence for horizontal pleiotropy (HP) was found (Egger intercept, *P* > 0.05; MR-PRESSO, *P* > 0.05). No significant association was detected between longevity and Pol III subunit expression in any other tissue, although a strong negative association could also be observed in the small intestine, where the number of *cis*-eQTLs available as instruments was low (Fig. 1B; Supplemental Table S1). Overall, MR analysis indicated that reduced expression of Pol III subunits in the adult adipose tissue is causally associated with human longevity.

### RP gene expression in the liver is associated with human longevity

**Figure 2.**
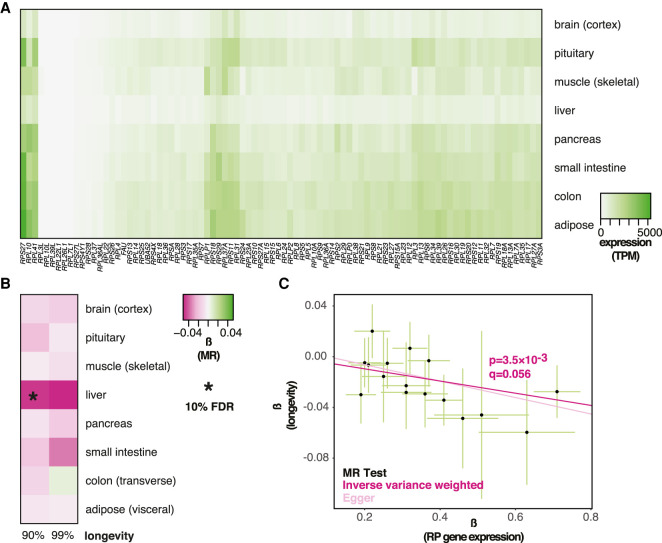
MR analysis of association between expression of RP genes and longevity. (*A*) Levels of mRNA coding for RPs in the indicated tissues are shown. (*B*) MR analysis was performed using RP gene expression in indicated tissues as exposures and longevity (survival beyond the 90th or 99th percentile) as outcomes. The values of β are given per tissue and the significant associations indicated as those that pass the 10% FDR threshold (accounting for eight tissues and two outcomes, i.e., 16 tests). (*C*) MR scatter plot of association between RP expression in liver and longevity (90th percentile), showing results of two MR methods. Each black point denotes a *cis*-eQTL; green bars show SEM. Longevity β is the natural log of the odds ratio, where lower values indicate a lower chance of surviving beyond the 90th percentile. Expression β is reported as NESs, with larger values indicating higher expression, and refers to expression of multiple genes.

We next examined the relationship between expression of over 80 human genes coding for RPs and longevity, as loss of function in RP genes can delay aging in yeast and worms (Kaeberlein et al. 2005; Chen et al. 2007; Chiocchetti et al. 2007; Curran and Ruvkun 2007; Hansen et al. 2007). RP gene expression was ubiquitous, and their overall expression levels varied across tissues (Fig. 2A). MR analysis, using RP gene *cis*-eQTLs as instruments and longevity as the outcome, revealed a negative, causal association with the likelihood of survival beyond the 90th percentile for RP expression in the liver, which was statistically significant at 10% FDR (β = −0.048 ± 0.016, *P* = 3.5 × 10^−3^, *q* = 0.056) (Fig. 2B,C; for per *cis*-eQTL effect sizes and gene annotation, see Supplemental Fig. S1B; for details of MR results for all tissues, see Supplemental Table S1). We found no evidence of HP (Egger intercept, *P* > 0.05; Mendelian randomization pleiotropy residual sum and outlier [MR-PRESSO] *P* > 0.05). Hence, in addition to reduced Pol III subunit expression in visceral adipose, we found evidence for a causal association where reduced expression of RP-coding genes in the liver promotes human longevity.

Some RPs have secondary, nonribosomal roles, and indeed, not all RP gene mutants are long-lived in model organisms (Kaeberlein et al. 2005; Chen et al. 2007; Chiocchetti et al. 2007; Curran and Ruvkun 2007; Hansen et al. 2007). For this reason, we additionally examined the relationship between the expression of a subset of RP genes in the liver and survival beyond the 90th percentile. Specifically, we looked at the RPs whose orthologs are implicated in longevity in model organisms and found that the variation in their expression in the liver was also negatively associated with longevity (β = −0.046 ± 0.018, *P* = 0.010) (Supplemental Fig. S2), confirming our initial findings.

### Human longevity is affected by Pol I subunit expression in skeletal muscle

**Figure 3.**
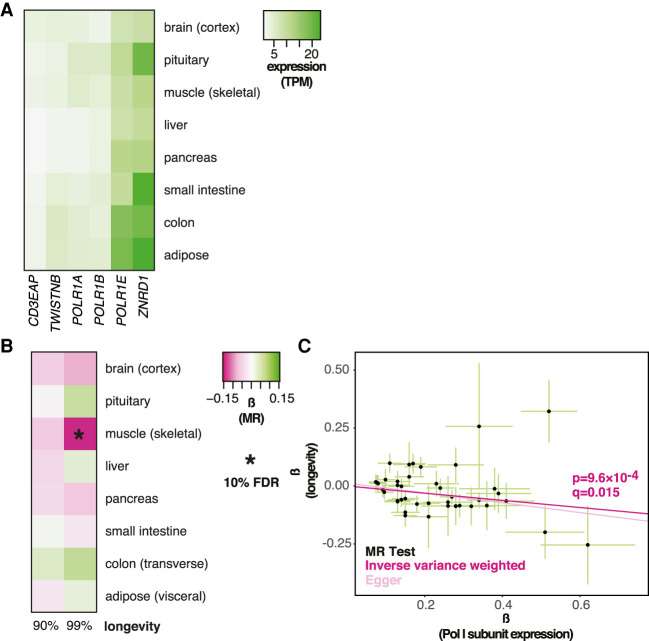
MR analysis of association between expression of Pol I–specific subunits and longevity. (*A*) Levels of mRNA coding for Pol I–specific subunits in the indicated tissues are shown. (*B*) MR analysis was performed using Pol I–specific subunit expression in the indicated tissues as exposures and longevity (survival beyond the 90th or 99th percentile) as outcomes. The values of β are given per tissue and the significant associations indicated as those that pass the 10% FDR threshold (accounting for eight tissues and two outcomes, i.e., 16 tests). (*C*) MR scatter plot of association between Pol I expression in skeletal muscle and longevity (99th percentile), showing results of two MR methods. Each black point denotes a *cis*-eQTL; green bars show SEM. Longevity β is the natural log of the odds ratio, where lower values indicate a lower chance of surviving beyond the 99th percentile. Expression β is reported as NESs, with larger values indicating higher expression, and refers to expression of multiple genes.

To date, Pol I has only been shown to affect aging in the fruit fly (Martínez et al. 2020). Similarly to Pol III and RP, the genes encoding the subunits unique to the Pol I complex were expressed ubiquitously, with levels of expression differing between tissues (Fig. 3A). MR analysis revealed a negative, causal association, with the likelihood of survival beyond the 99th percentile in skeletal muscle that was statistically significant at 10% FDR (β = −0.15 ± 0.047, *P* = 9.6 × 10^−4^, *q* = 0.015) (Fig. 3B,C; for per *cis*-eQTL effect sizes and gene annotation, see Supplemental Fig. S1C; for details of MR results for all tissues, see Supplemental Table S1). Again, this organ was distinct from those where Pol III subunit or RP gene expression was relevant for longevity. In this case, we found some evidence of HP (Egger intercept, *P* > 0.05; MR-PRESSO *P* = 3 × 10^−3^), indicating that the genetic variants used in MR may not affect longevity solely through their effect on Pol I subunit expression in muscle or, alternatively, that the relationship between expression and longevity may not be linear, as indeed is the case in the fruit fly (Martínez et al. 2020). Using MR-PRESSO to correct for outliers, the negative association between muscle expression and longevity remained significant (corrected for one outlier, β = −0.18, *P* = 1.4 × 10^−4^, no significant distortion). Overall, we found evidence that the expression of Pol I subunits in skeletal muscle is causally associated with human longevity.

### Effects on longevity are unlikely to be explained by susceptibility to a specific age-related disease and are not observed for Pol II–specific subunit expression

The longevity phenotype captures the relative time of death but not the cause of death. For this reason, the link between expression of genes required for protein synthesis and longevity could be explained by the genes’ effects on one or few age-related diseases rather than on organismal aging more broadly. For example, if RP expression solely affected cancer susceptibility and progression, but not aging more broadly, it may still have a noticeable effect on longevity per se. To examine this, we performed MR analysis using either the Pol III or Pol I subunit or RP gene expression as exposures and a range of age-related diseases as outcomes. We limited the analysis to tissues in which a significant effect on longevity was observed for a specific gene set, namely, Pol III in visceral adipose, RP in liver, and Pol I in skeletal muscle. The associations that were significant and passed the 10% FDR threshold (accounting for 10 tests per exposure) are shown in Table 1 (for full analysis, see Supplemental Table S2). The only strong associations identified were for Pol I subunit expression in skeletal muscle, where increased expression was associated with a reduced risk of coronary heart disease and a decreased risk of amyotrophic lateral sclerosis (ALS) but an increased risk of Alzheimer's disease. Note that in models of Alzheimer's disease and ALS, protein synthetic machinery has been implicated in diseases pathophysiology, albeit cell autonomously (Meier et al. 2016; Moens et al. 2019; Yamada et al. 2019). The complex modulation of disease susceptibility observed for Pol I skeletal muscle expression makes it unlikely, albeit possible, that the effects on longevity were owing to a differential susceptibility to a single disease, such as Alzheimer's. Overall, our analysis did not provide substantial evidence that any of the longevity effects we observed could be explained by altered susceptibility to a single disease, indicating they may be caused by a broad effect on aging itself.

**Table 1.**
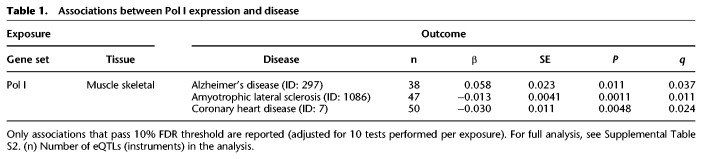
Associations between Pol I expression and disease

We also examined several anthropometric and developmental traits, as well as smoking as a common confounder, finding no associations that passed the 10% FDR threshold (Supplemental Table S2).

Pols I and III are more specifically involved in the production of translation machinery, whereas Pol II is required for expression of all coding, messenger RNAs (Vannini and Cramer 2012). To further examine if longevity was associated specifically with provision of translation machinery rather than general transcription, we performed MR analysis for Pol II–specific subunits. We found no significant associations with longevity for Pol II subunit expression in any tissue at 10% FDR (Supplemental Table S1), indicating that longevity is specifically associated with RNA Pols that are dedicated to the production of the translation machinery.

## Discussion

The biogenesis of the translation machinery is an essential cellular process required for cell and organismal growth (Grewal et al. 2007; Marygold et al. 2007; Marshall et al. 2012). Indeed, mutations in human genes that are key to this process can be linked to a number of developmental diseases (Mills and Green 2017). In contrast, in this study we provide evidence that a reduced expression of these genes in particular tissues in adult humans is causally linked to human longevity. The magnitude of the reduction in expression that favors longevity corresponds in scale to the natural, genetically encoded variation observed in human populations. Hence, it is likely that the relative reduction in biogenesis of the translation machinery that promotes longevity has substantially less effect on overall cellular capacity for translation than do disease-causing mutations. The contrast between the essential role of the translation machinery in development and its proaging, detrimental effect on the adult is consistent with evolutionary theories of aging, specifically antagonistic pleiotropy (Williams 1957): The rate of production of translation machinery is selected for optimal growth and reproduction even if it comes at a cost of accelerated aging later in life.

Our analyses indicate that Pol I, Pol III, or RPs limit longevity from different adult tissues, namely, muscle, adipose, and liver, respectively. This is akin to tissue specificity of ribosomopathies that may be explained by different protein synthesis requirements for different cells or cellular functions (Mills and Green 2017; Farley-Barnes et al. 2019), and would imply that protein synthesis drives aging from (at least) each of these three tissues. Indeed, previous work has shown that protein translation capacity plays an important role in the biology of each tissue; for example, Pol III plays an important role in adipocyte differentiation (Chen et al. 2018); tight control of ribosome biogenesis is likely to underlie the liver's daily cycles of protein synthesis (Sinturel et al. 2017); and reduced hepatic protein synthesis is observed in long-lived mouse models (Thompson et al. 2016), whereas rDNA transcription by Pol I appears pivotal in the aging of skeletal muscle (Kirby et al. 2015). Each of the three tissues plays an important role in whole-animal metabolism. Although the link between gene expression and longevity is observed in specific tissues, the underlying mechanisms may involve tissue-nonautonomous effects such as those mediated by metabolic changes or endocrine signaling. Indeed, altering translation in specific tissues during development can have nonautonomous, endocrine effects on organismal growth in *Drosophila* (Lin et al. 2011; Marshall et al. 2012). Additionally, a recent report indicates that improved fidelity of translation can also promote longevity in the fruit fly (Martinez-Miguel et al. 2021).

Ethical considerations and costs of clinical trials severely limit the possibilities to investigate fundamental mechanisms of aging in humans. Numerous studies have shown that biogenesis of translational machinery drives aging in a number of experimental models, including yeast, nematode worms, and fruit flies (Kaeberlein et al. 2005; Chen et al. 2007; Chiocchetti et al. 2007; Curran and Ruvkun 2007; Hansen et al. 2007; Filer et al. 2017; Tiku et al. 2017; Martínez et al. 2020). We believe our study to be the first to show that this process is likely to be similarly relevant in the context of human aging. The paradigm we established leverages natural human variation in gene expression and can assess subtle, tissue-specific effects on longevity. This approach is especially useful for essential genes, in which complete knockouts are very rare or not observed owing to a severe impact on development, and overcomes the issues of assigning functional consequences to coding sequence variants. One drawback of our method is that the causal estimate β is essentially unit-less, as we are using the variation in mRNA expression of multiple genes as a proxy for the activity of the complex formed by the genes’ protein products. However, the method does allow us to identify significant associations and, importantly, define their direction. The application of MR and the utilization of public data resources make it possible for future studies to apply this paradigm to investigate the relevance of other pathways that have been implicated in aging in animal models to humans. Finally, our work indicates that further studies of the link between translation machinery and aging are warranted and may result in interventions and treatments for human aging and age-related conditions.

## Methods

### Instrument selection

The human genes encoding the specific subunits of Pol I, II, and III or RPs were identified based on published literature (Vannini and Cramer 2012; Khatter et al. 2015). We identified *cis*-eQTLs for each gene using GTEx version 8 (The GTEx Consortium 2020) and combined them together based on their belonging to Pol III, ribosome, Pol I, or Pol II. The human orthologs of the genes implicated in yeast or worm aging were obtained from the *Saccharomyces* Genome Database and from WormBase. The lists of genes per complex are provided in Supplemental Table S3.

In short, *cis*-eQTLs analysis was performed by GTEx consortium using FastQTL (Ongen et al. 2016). Based on 838 donors with whole-genome sequencing available, genes were included in tissue-specific eQTL analysis if expression thresholds of >0.1 TPM and six or more reads were detected in at least 20% of samples. For each gene, variants with a MAF > 0.01 in the 838 donors and located in ±1-Mb window around the transcription start site (TSS) were considered for *cis*-eQTL analysis. GTEx contains up to 17% of individuals with non-European or admixed ancestry (Gay et al. 2020). The association analyses between SNPs and gene expression were adjusted for ancestry by including the first five principal components from a principal component analysis based on genetic similarity between the samples.

The GTEx tissue dashboard was used to query for single tissue *cis*-eQTLs in eight tissues of interest: (1) adipose (visceral), (2) colon (transverse), (3) small intestine, (4) pancreas, (5) liver, (6) muscle (skeletal), (7) pituitary, and (8) brain (cortex). Query results for all significantly associated *cis*-eQTLs (FDR < 0.05) containing GENCODE ID, gene symbol, variant ID, dbSNP ID, *P*-value, and effect size were downloaded for subsequent analyses. The *cis*-eQTL associations were assessed by the GTEx investigators using linear regression, implemented in FastQTL (Ongen et al. 2016) after removal of low expressing genes. Effect sizes for *cis*-eQTLs are reported as normalized effect sizes (NESs) calculated as effect of the alternative allele (ALT) relative to the reference allele (REF). NESs are computed in a normalized space as the slope in the linear regression; any noise from potential residual variation in the relative NES between genes is unlikely to result in false-positive associations as it is unlikely to be linked to the outcome.

Effect allele frequencies were obtained from gnomAD v2.1.1 for European (Non-Finnish) ancestry samples. To ensure independence of instruments, linkage disequilibrium (LD) pruning was performed for each gene using the 1000 genomes reference panel for European ancestry samples and excluding weaker instrument (determined as larger *P*-value for eQTL) in pairings exceeding *r*² = 0.1. The lists of all *cis*-eQTLs that were fed into the MR analysis are provided in Supplemental Table S4, including details of their genomic location and the affected gene.

### Tissue-specific two-sample MR

We used R version 3.6.1 (R Core Team 2017) and the TwoSampleMR package version 0.5.3 (Hemani et al. 2018) to perform two-sample MR separately for the eight tissues of interest.

To assess causal effects of sets of genes coding for either subunits specific to Pol I, II, or III or genes encoding RPs on human longevity, we obtained the summary statistics of a meta-analysis of genome-wide association studies, where cases were defined as persons living to an age above the 90th of 99th percentile of their cohort, based on cohort life tables (Deelen et al. 2019). The meta-analysis was based on 11,262 case and 25,483 control samples of European ancestry from 18 studies. Longevity meta-analysis summary statistics were kindly provided by J. Deelen.

After data harmonization between tissue-specific pooled *cis*-eQTL instruments per gene set (Pol I, II, or III or RPs) and outcome meta-analysis of life span, MR estimates were obtained using MR methods inverse variance weighted (IVW) (Burgess et al. 2013), MR Egger regression, and weighted median. Harmonized data are provided in Supplemental Table S5.

We also investigated whether associations of Pol I or III and RP with longevity were mediated by effects on a single disease or risk factor. We included common age-related diseases and smoking. GWAS summary statistics were extracted from the largest publicly available study. The antagonistic pleiotropy theory states aging is related to mutations that are beneficial for early fitness but harmful for the organism in later life. To assess whether we see such pleiotropic effects, we also included some developmental and anthropometric traits. MR on these other outcomes was performed with MR-Base online (Hemani et al. 2018). Please find the detailed description of studies included for age-related disease outcomes, as well as developmental and anthropometric trait outcomes, in Supplemental Table S6.

### HP analysis

The presence of HP was investigated using MR Egger estimates (Bowden et al. 2015), as well as MR-PRESSO (Verbanck et al. 2018). In short, the intercept of a MR Egger regression gives an estimate of the average pleiotropic effect across all instruments. MR-PRESSO will also test for significant global HP and will additionally test individual instruments. This allows the detection of outliers, their removal, and the estimation of distortion in causal effect estimates owing to inclusion of instruments with significant pleiotropic effects.

### Multiple testing correction and correlation analysis

The *P*-value threshold for 10% FDR was calculated using the Benjamini and Hochberg procedure (Benjamini and Hochberg 1995) to account for the number of tissues (eight) and the two case definitions (for longevity) or the number of outcomes (for disease or developmental and anthropometric traits). This means that for the longevity analysis, we accounted for 16 tests, whereas for disease or developmental and the anthropometric traits, we accounted for 10 tests. We calculated the *q*-values using the *qvalue* package version 2.16.0 in R with conservative parameters that approximate the Benjamini and Hochberg procedure (lambda = 0) accounting for the same numbers of tests as indicated above (Storey et al. 2004).

### Expression data

GTEx transcript abundance data were obtained from the Human Protein Atlas version 19.3 (Uhlén et al. 2005) for expression in adipose tissue, cerebral cortex, colon, liver, pancreas, pituitary gland, skeletal muscle, and small intestine.

## Supplementary Material

Supplemental Material
