# Supplementary material for: Mendelian randomization analyses implicate biogenesis of translation machinery in human aging
Source: Genome Res. 2022 Feb;32(2):258–65. doi: 10.1101/gr.275636.121 (PMC8805714; doi:10.1101/gr.275636.121)
Supplement: Supplemental Material [file supp_32_2_258__DC1.html]

Mendelian randomization analyses implicate biogenesis of translation machinery in human aging — Supplemental Material 

# Mendelian randomization analyses implicate biogenesis of translation machinery in human aging

## Supplemental Material

- Supplemental\_Fig\_S1.pdf
- Supplemental\_Fig\_S2.pdf
- Supplemental\_Table\_S1.xlsx
- Supplemental\_Table\_S2.xlsx
- Supplemental\_Table\_S3.xlsx
- Supplemental\_Table\_S4.xlsx
- Supplemental\_Table\_S5.xlsx
- Supplemental\_Table\_S6.xlsx
