# Supplementary material for: Mendelian randomization analyses implicate biogenesis of translation machinery in human aging
Source: Genome Res. 2022 Feb;32(2):258–65. doi: 10.1101/gr.275636.121 (PMC8805714; doi:10.1101/gr.275636.121)
Supplement: Supplemental Material [file supp_gr.275636.121_Supplemental_Fig_S1_.pdf]

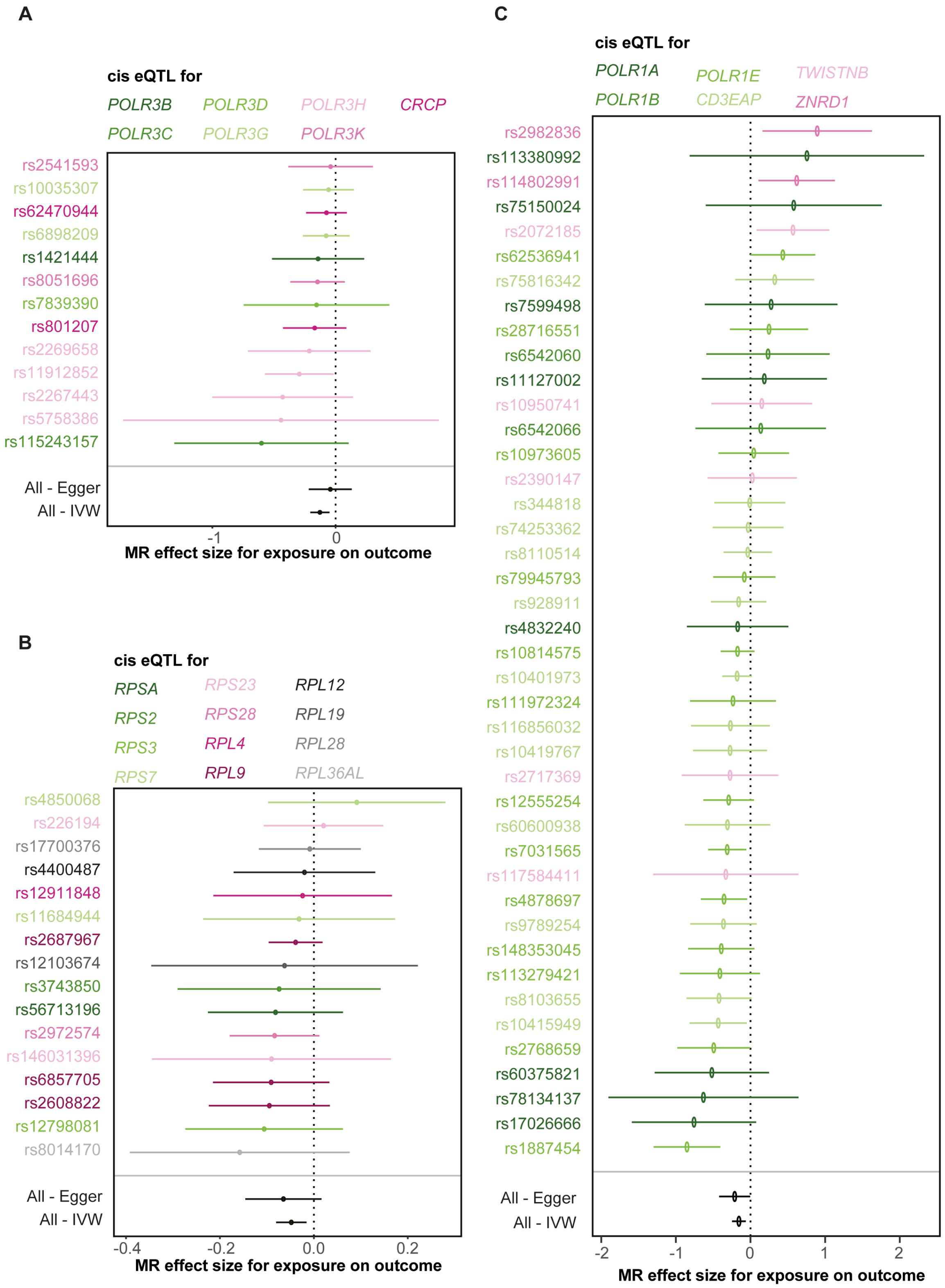

Supplemental Figure 1 Forest Plots.

Forest plots showing effect of exposure on outcome for individual cis-eQTL, where the gene associated with the cis-eQTL is colour-coded, for **A** Pol III subunit expression in visceral adipose and 90th percentile longevity, **B** RP gene expression in liver and 90th percentile longevity and **C** Pol I subunit expression in skeletal muscle and 99th percentile longevity, determined by MR.
