# Supplementary material for: Mendelian randomization analyses implicate biogenesis of translation machinery in human aging
Source: Genome Res. 2022 Feb;32(2):258–65. doi: 10.1101/gr.275636.121 (PMC8805714; doi:10.1101/gr.275636.121)
Supplement: Supplemental Material [file supp_gr.275636.121_Supplemental_Fig_S2.pdf]

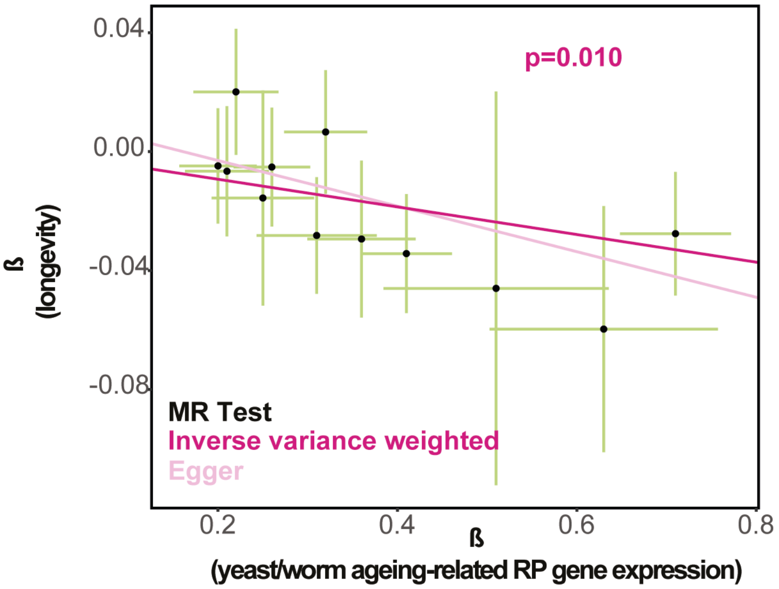

Supplemental Figure 2 Mendelian Randomisation for a subset of RPs.
MR scatter plots of association between RP expression in liver, for RP genes whose orthologues were experimentally shown to have a role in ageing in yeast or worms, and longevity (90th percentile) showing results of two MR methods (IVW ß=-0.046±0.018, p=0.010). Each black point denotes a cis-eQTL; green bars show SEM. This subset includes 12 out of 16 cis-eQTLs shown in Figure 2C. Longevity ß is the natural log of the odds ratio where lower values indicate lower chance of surviving beyond the 90th percentile. Expression ß is reported as reported as NES with larger values indicating higher expression and refers to expression of multiple genes.
